# Supplementary material for: Unveiling the Bioactive Potential of the Invasive Jellyfish Phyllorhiza punctata Through Integrative Transcriptomic and Proteomic Analyses
Source: Biomolecules. 2025 Aug 4;15(8):1121. doi: 10.3390/biom15081121 (PMC12383608; doi:10.3390/biom15081121)
Supplement: Supplementary file 1 [file biomolecules-15-01121-s001.zip › Suplementary Material/FileS3/PEPFOLD4/XP_065067317_326/clusters.html]

 PEP-FOLD clusters


Download archive of all models  here   

|  |  |  |
| --- | --- | --- |
| # | file | sOPEP |
| # cluster 1 - Size: 29 |  |  |
|  | XP\_065067317\_326-model1 | -19.8032 |
|  | XP\_065067317\_326-model1.1 | -19.7623 |
|  | XP\_065067317\_326-model1.2 | -19.7541 |
|  | XP\_065067317\_326-model1.3 | -19.7298 |
|  | XP\_065067317\_326-model1.4 | -19.7291 |
|  | XP\_065067317\_326-model1.5 | -19.7165 |
|  | XP\_065067317\_326-model1.6 | -19.6938 |
|  | XP\_065067317\_326-model1.7 | -19.6935 |
|  | XP\_065067317\_326-model1.8 | -19.6582 |
|  | XP\_065067317\_326-model1.9 | -19.6363 |
|  | XP\_065067317\_326-model1.10 | -19.6334 |
|  | XP\_065067317\_326-model1.11 | -19.6273 |
|  | XP\_065067317\_326-model1.12 | -19.6253 |
|  | XP\_065067317\_326-model1.13 | -19.6097 |
|  | XP\_065067317\_326-model1.14 | -19.6097 |
|  | XP\_065067317\_326-model1.15 | -19.5446 |
|  | XP\_065067317\_326-model1.16 | -19.5419 |
|  | XP\_065067317\_326-model1.17 | -19.5363 |
|  | XP\_065067317\_326-model1.18 | -19.5291 |
|  | XP\_065067317\_326-model1.19 | -19.484 |
|  | XP\_065067317\_326-model1.20 | -19.484 |
|  | XP\_065067317\_326-model1.21 | -19.4771 |
|  | XP\_065067317\_326-model1.22 | -19.4561 |
|  | XP\_065067317\_326-model1.23 | -19.4283 |
|  | XP\_065067317\_326-model1.24 | -19.4202 |
|  | XP\_065067317\_326-model1.25 | -19.416 |
|  | XP\_065067317\_326-model1.26 | -19.3065 |
|  | XP\_065067317\_326-model1.27 | -19.2801 |
|  | XP\_065067317\_326-model1.28 | -17.9675 |
| # cluster 2 - Size: 1 |  |  |
|  | XP\_065067317\_326-model2 | -15.5335 |
| # cluster 3 - Size: 35 |  |  |
|  | XP\_065067317\_326-model3 | -15.1329 |
|  | XP\_065067317\_326-model3.1 | -15.0463 |
|  | XP\_065067317\_326-model3.2 | -15.0129 |
|  | XP\_065067317\_326-model3.3 | -14.9752 |
|  | XP\_065067317\_326-model3.4 | -14.9539 |
|  | XP\_065067317\_326-model3.5 | -14.9383 |
|  | XP\_065067317\_326-model3.6 | -14.8917 |
|  | XP\_065067317\_326-model3.7 | -14.8897 |
|  | XP\_065067317\_326-model3.8 | -14.8204 |
|  | XP\_065067317\_326-model3.9 | -14.789 |
|  | XP\_065067317\_326-model3.10 | -14.7669 |
|  | XP\_065067317\_326-model3.11 | -14.7386 |
|  | XP\_065067317\_326-model3.12 | -14.7357 |
|  | XP\_065067317\_326-model3.13 | -14.7214 |
|  | XP\_065067317\_326-model3.14 | -14.6881 |
|  | XP\_065067317\_326-model3.15 | -14.6881 |
|  | XP\_065067317\_326-model3.16 | -14.6815 |
|  | XP\_065067317\_326-model3.17 | -14.6138 |
|  | XP\_065067317\_326-model3.18 | -14.5861 |
|  | XP\_065067317\_326-model3.19 | -14.4567 |
|  | XP\_065067317\_326-model3.20 | -14.4498 |
|  | XP\_065067317\_326-model3.21 | -14.419 |
|  | XP\_065067317\_326-model3.22 | -14.4071 |
|  | XP\_065067317\_326-model3.23 | -14.3961 |
|  | XP\_065067317\_326-model3.24 | -14.2953 |
|  | XP\_065067317\_326-model3.25 | -14.1959 |
|  | XP\_065067317\_326-model3.26 | -14.1959 |
|  | XP\_065067317\_326-model3.27 | -14.1784 |
|  | XP\_065067317\_326-model3.28 | -14.1473 |
|  | XP\_065067317\_326-model3.29 | -14.1381 |
|  | XP\_065067317\_326-model3.30 | -14.1303 |
|  | XP\_065067317\_326-model3.31 | -14.0973 |
|  | XP\_065067317\_326-model3.32 | -14.0855 |
|  | XP\_065067317\_326-model3.33 | -14.0735 |
|  | XP\_065067317\_326-model3.34 | -13.8563 |
| # cluster 4 - Size: 13 |  |  |
|  | XP\_065067317\_326-model4 | -14.3649 |
|  | XP\_065067317\_326-model4.1 | -14.2189 |
|  | XP\_065067317\_326-model4.2 | -14.2025 |
|  | XP\_065067317\_326-model4.3 | -14.1548 |
|  | XP\_065067317\_326-model4.4 | -14.1548 |
|  | XP\_065067317\_326-model4.5 | -14.1548 |
|  | XP\_065067317\_326-model4.6 | -14.0913 |
|  | XP\_065067317\_326-model4.7 | -14.0773 |
|  | XP\_065067317\_326-model4.8 | -14.0209 |
|  | XP\_065067317\_326-model4.9 | -14.0209 |
|  | XP\_065067317\_326-model4.10 | -13.8714 |
|  | XP\_065067317\_326-model4.11 | -13.8627 |
|  | XP\_065067317\_326-model4.12 | -12.9589 |
| # cluster 5 - Size: 5 |  |  |
|  | XP\_065067317\_326-model5 | -14.3099 |
|  | XP\_065067317\_326-model5.1 | -14.2937 |
|  | XP\_065067317\_326-model5.2 | -13.6557 |
|  | XP\_065067317\_326-model5.3 | -13.2968 |
|  | XP\_065067317\_326-model5.4 | -11.4089 |
| # cluster 6 - Size: 6 |  |  |
|  | XP\_065067317\_326-model6 | -14.2878 |
|  | XP\_065067317\_326-model6.1 | -14.2128 |
|  | XP\_065067317\_326-model6.2 | -14.1042 |
|  | XP\_065067317\_326-model6.3 | -14.0096 |
|  | XP\_065067317\_326-model6.4 | -13.9757 |
|  | XP\_065067317\_326-model6.5 | -13.9188 |
| # cluster 7 - Size: 1 |  |  |
|  | XP\_065067317\_326-model7 | -11.3567 |
| # cluster 8 - Size: 4 |  |  |
|  | XP\_065067317\_326-model8 | -10.6052 |
|  | XP\_065067317\_326-model8.1 | -10.5388 |
|  | XP\_065067317\_326-model8.2 | -10.4804 |
|  | XP\_065067317\_326-model8.3 | -10.4231 |
| # cluster 9 - Size: 3 |  |  |
|  | XP\_065067317\_326-model9 | -10.5075 |
|  | XP\_065067317\_326-model9.1 | -10.4774 |
|  | XP\_065067317\_326-model9.2 | -10.2383 |
| # cluster 10 - Size: 2 |  |  |
|  | XP\_065067317\_326-model10 | -10.4709 |
|  | XP\_065067317\_326-model10.1 | -10.4099 |
| # cluster 11 - Size: 1 |  |  |
|  | XP\_065067317\_326-model11 | -5.20407 |
|  |  |  |
